# Supplementary material for: Selection of Gut-Resistant Bacteria and Construction of Microbial Consortia for Improving Gluten Digestion under Simulated Gastrointestinal Conditions
Source: Nutrients. 2021 Mar 19;13(3):992. doi: 10.3390/nu13030992 (PMC8003469; doi:10.3390/nu13030992)
Supplement: Supplementary file 1 [file nutrients-13-00992-s001.zip › Supplementary Figure S1.pdf]

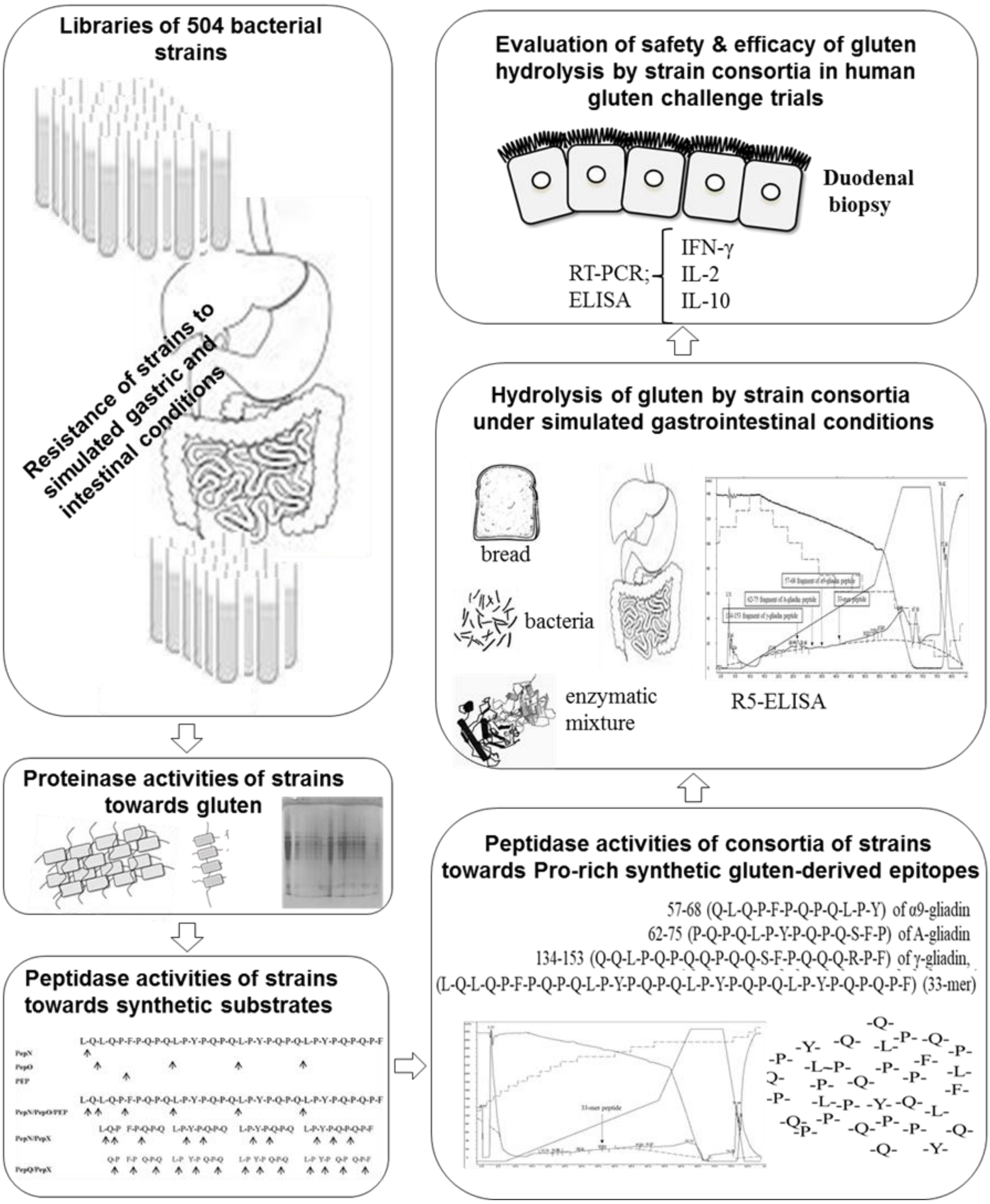

Libraries of 504 bacterial strains
Resistance of strains to simulated gastric and intestinal conditions
Proteinase activities of strains towards gluten
Peptidase activities of strains towards synthetic substrates
L-Q-L-Q-P-F-P-Q-P-Q-L-P-Y-P-Q-P-Q-L-P-Y-P-Q-P-Q-L-P-Y-P-Q-P-Q-P-F
Peptidase activities of consortia of strains towards Pro-rich synthetic gluten-derived epitopes
57-68 (Q-L-Q-P-F-P-Q-P-Q-L-P-Y) of α9-gliadin
62-75 (P-Q-P-Q-L-P-Y-P-Q-P-Q-S-F-P) of A-gliadin
134-153 (Q-Q-L-P-Q-P-Q-Q-P-Q-Q-S-F-P-Q-Q-Q-R-P-F) of γ-gliadin,
(L-Q-L-Q-P-F-P-Q-P-Q-L-P-Y-P-Q-P-Q-L-P-Y-P-Q-P-Q-L-P-Y-P-Q-P-Q-P-F) (33-mer)
Hydrolysis of gluten by strain consortia under simulated gastrointestinal conditions
bread
bacteria
enzymatic mixture
R5-ELISA
Evaluation of safety & efficacy of gluten hydrolysis by strain consortia in human gluten challenge trials
Duodenal biopsy
RT-PCR;
ELISA
IFN-γ
IL-2
IL-10
