# Supplementary material for: Selection of Gut-Resistant Bacteria and Construction of Microbial Consortia for Improving Gluten Digestion under Simulated Gastrointestinal Conditions
Source: Nutrients. 2021 Mar 19;13(3):992. doi: 10.3390/nu13030992 (PMC8003469; doi:10.3390/nu13030992)
Supplement: Supplementary file 1 [file nutrients-13-00992-s001.zip › Supplementary Figure S3.pdf]

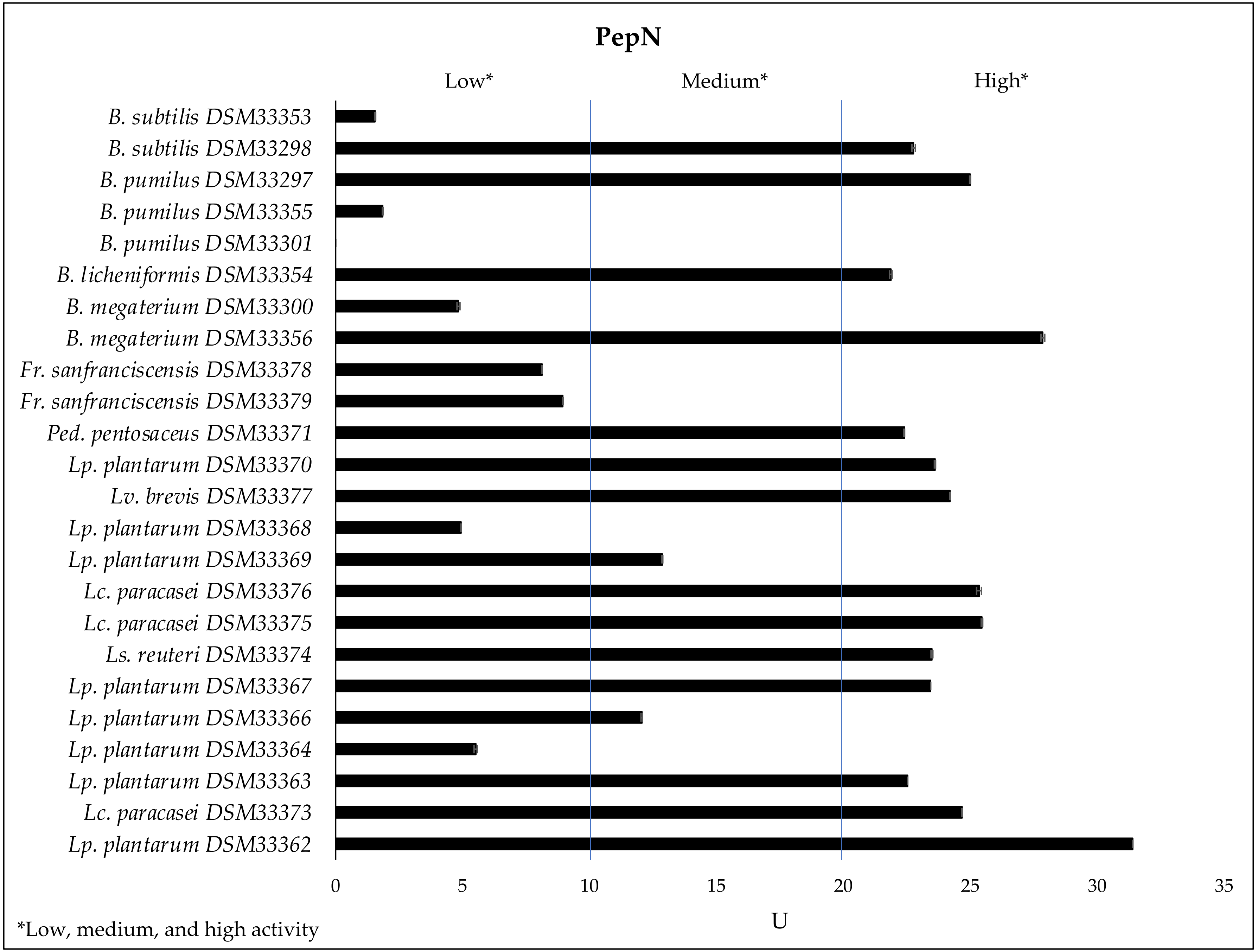
PepN
Low*
Medium*
High*
B. subtilis DSM33353
B. subtilis DSM33298
B. pumilus DSM33297
B. pumilus DSM33355
B. pumilus DSM33301
B. licheniformis DSM33354
B. megaterium DSM33300
B. megaterium DSM33356
Fr. sanfranciscensis DSM33378
Fr. sanfranciscensis DSM33379
Ped. pentosaceus DSM33371
Lp. plantarum DSM33370
Lv. brevis DSM33377
Lp. plantarum DSM33368
Lp. plantarum DSM33369
Lc. paracasei DSM33376
Lc. paracasei DSM33375
Ls. reuteri DSM33374
Lp. plantarum DSM33367
Lp. plantarum DSM33366
Lp. plantarum DSM33364
Lp. plantarum DSM33363
Lc. paracasei DSM33373
Lp. plantarum DSM33362
0
5
10
15
20
25
30
35
U
*Low, medium, and high activity

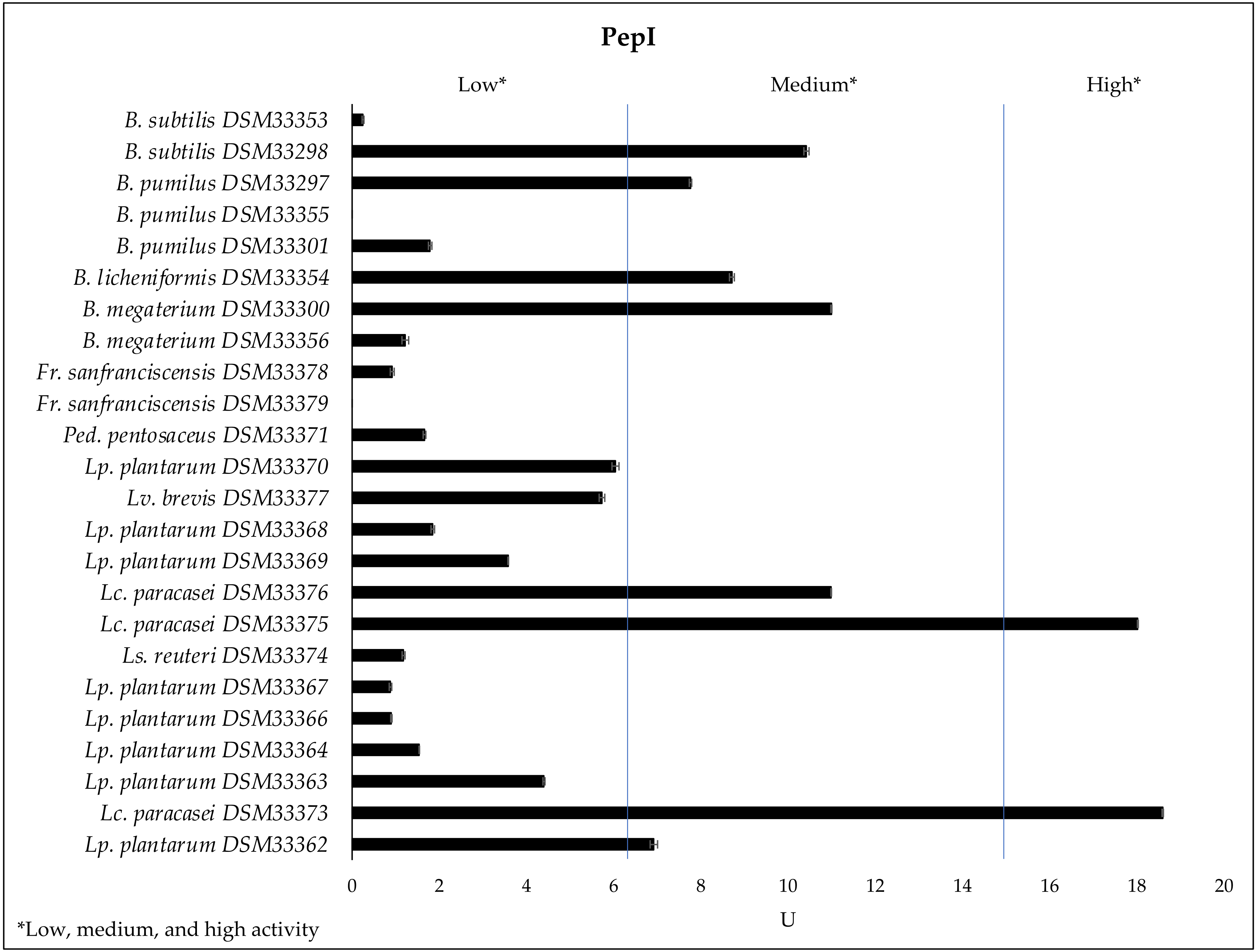
PepI
Low*
Medium*
High*
B. subtilis DSM33353
B. subtilis DSM33298
B. pumilus DSM33297
B. pumilus DSM33355
B. pumilus DSM33301
B. licheniformis DSM33354
B. megaterium DSM33300
B. megaterium DSM33356
Fr. sanfranciscensis DSM33378
Fr. sanfranciscensis DSM33379
Ped. pentosaceus DSM33371
Lp. plantarum DSM33370
Lv. brevis DSM33377
Lp. plantarum DSM33368
Lp. plantarum DSM33369
Lc. paracasei DSM33376
Lc. paracasei DSM33375
Ls. reuteri DSM33374
Lp. plantarum DSM33367
Lp. plantarum DSM33366
Lp. plantarum DSM33364
Lp. plantarum DSM33363
Lc. paracasei DSM33373
Lp. plantarum DSM33362
0
2
4
6
8
10
12
14
16
18
20
U
*Low, medium, and high activity

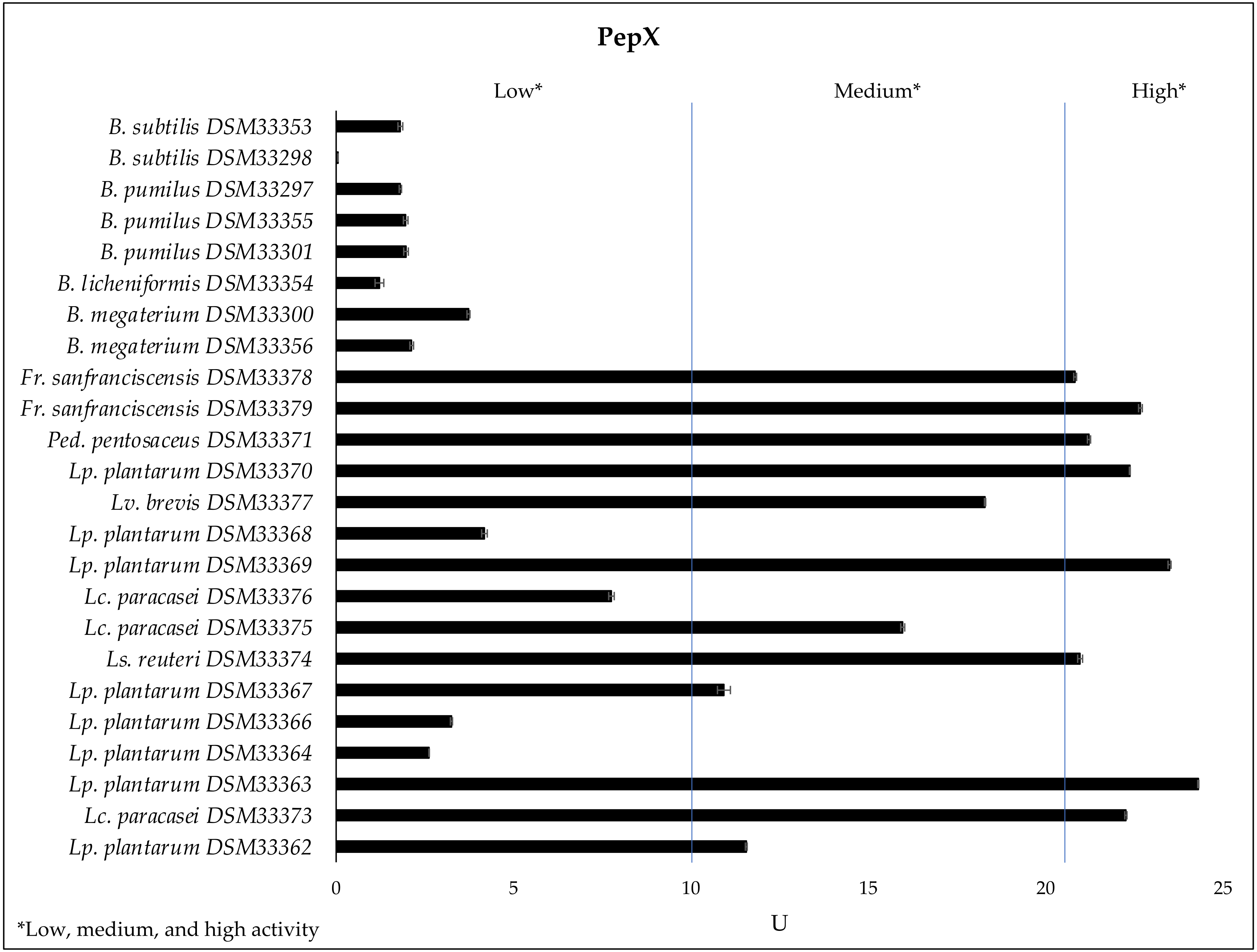
PepX
Low*
Medium*
High*
B. subtilis DSM33353
B. subtilis DSM33298
B. pumilus DSM33297
B. pumilus DSM33355
B. pumilus DSM33301
B. licheniformis DSM33354
B. megaterium DSM33300
B. megaterium DSM33356
Fr. sanfranciscensis DSM33378
Fr. sanfranciscensis DSM33379
Ped. pentosaceus DSM33371
Lp. plantarum DSM33370
Lv. brevis DSM33377
Lp. plantarum DSM33368
Lp. plantarum DSM33369
Lc. paracasei DSM33376
Lc. paracasei DSM33375
Ls. reuteri DSM33374
Lp. plantarum DSM33367
Lp. plantarum DSM33366
Lp. plantarum DSM33364
Lp. plantarum DSM33363
Lc. paracasei DSM33373
Lp. plantarum DSM33362
0
5
10
15
20
25
U
*Low, medium, and high activity

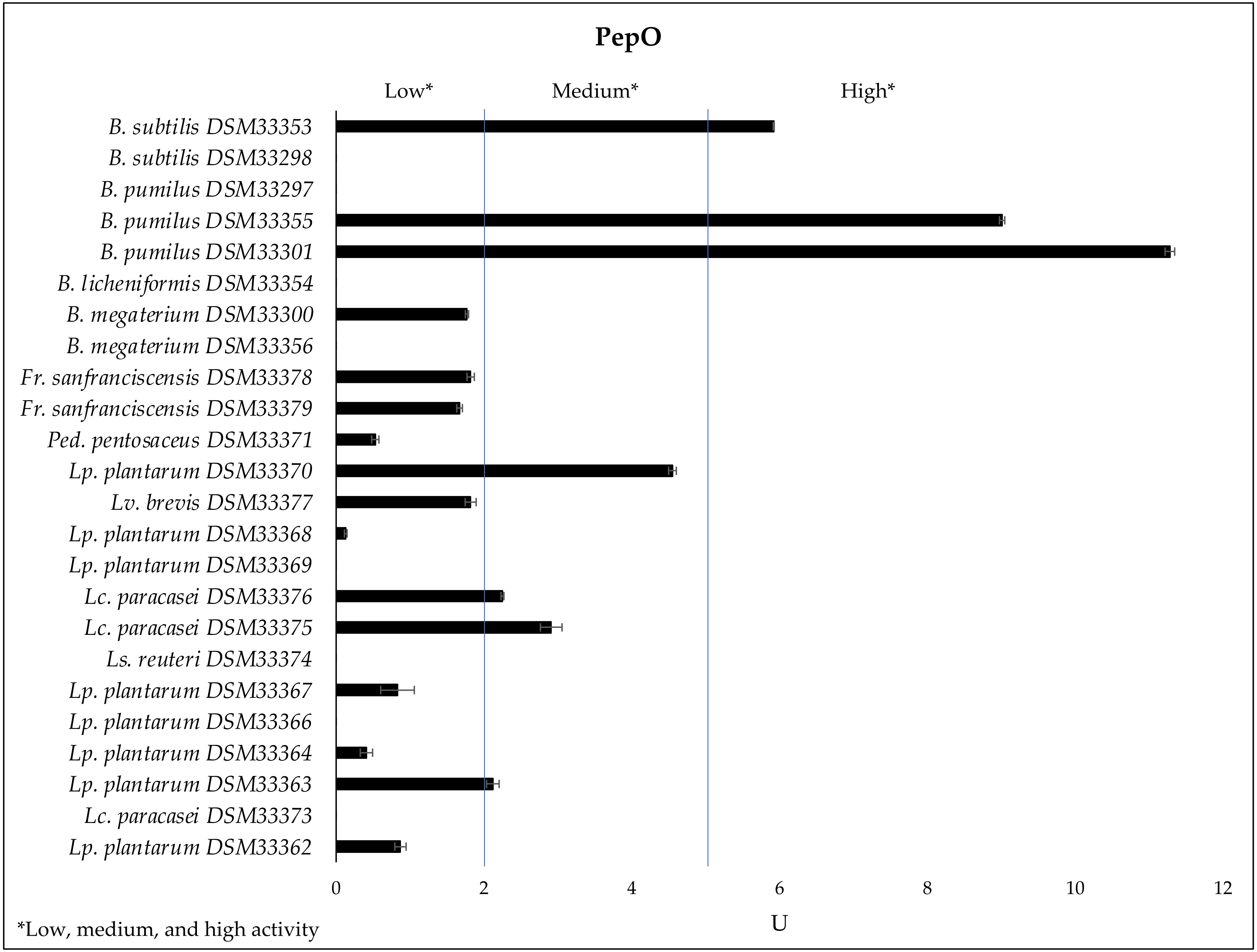
PepO
Low*
Medium*
High*
B. subtilis DSM33353
B. subtilis DSM33298
B. pumilus DSM33297
B. pumilus DSM33355
B. pumilus DSM33301
B. licheniformis DSM33354
B. megaterium DSM33300
B. megaterium DSM33356
Fr. sanfranciscensis DSM33378
Fr. sanfranciscensis DSM33379
Ped. pentosaceus DSM33371
Lp. plantarum DSM33370
Lv. brevis DSM33377
Lp. plantarum DSM33368
Lp. plantarum DSM33369
Lc. paracasei DSM33376
Lc. paracasei DSM33375
Ls. reuteri DSM33374
Lp. plantarum DSM33367
Lp. plantarum DSM33366
Lp. plantarum DSM33364
Lp. plantarum DSM33363
Lc. paracasei DSM33373
Lp. plantarum DSM33362
0
2
4
6
8
10
12
U
*Low, medium, and high activity

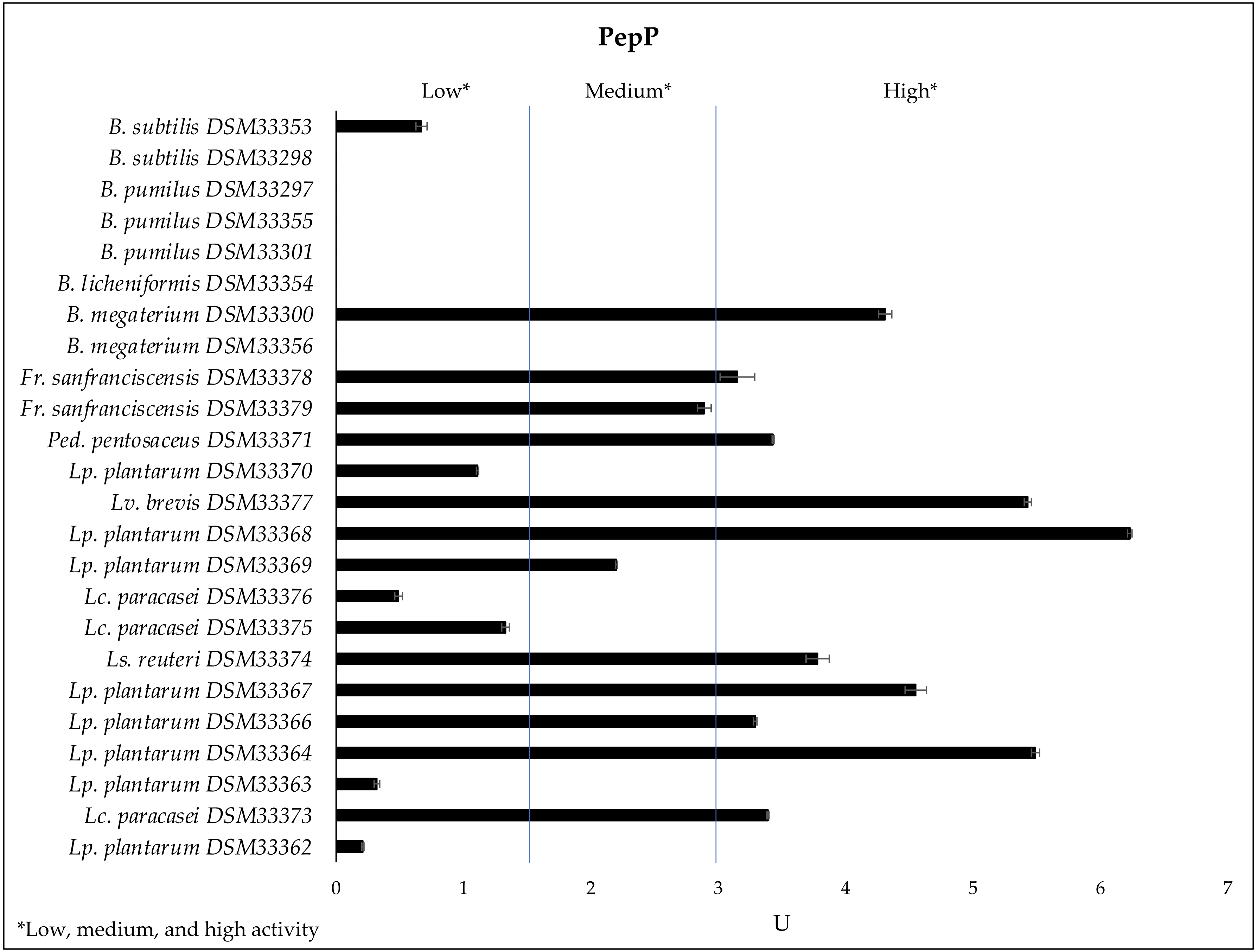
PepP
Low*
Medium*
High*
B. subtilis DSM33353
B. subtilis DSM33298
B. pumilus DSM33297
B. pumilus DSM33355
B. pumilus DSM33301
B. licheniformis DSM33354
B. megaterium DSM33300
B. megaterium DSM33356
Fr. sanfranciscensis DSM33378
Fr. sanfranciscensis DSM33379
Ped. pentosaceus DSM33371
Lp. plantarum DSM33370
Lv. brevis DSM33377
Lp. plantarum DSM33368
Lp. plantarum DSM33369
Lc. paracasei DSM33376
Lc. paracasei DSM33375
Ls. reuteri DSM33374
Lp. plantarum DSM33367
Lp. plantarum DSM33366
Lp. plantarum DSM33364
Lp. plantarum DSM33363
Lc. paracasei DSM33373
Lp. plantarum DSM33362
0
1
2
3
4
5
6
7
U
*Low, medium, and high activity
