# Supplementary material for: Selection of Gut-Resistant Bacteria and Construction of Microbial Consortia for Improving Gluten Digestion under Simulated Gastrointestinal Conditions
Source: Nutrients. 2021 Mar 19;13(3):992. doi: 10.3390/nu13030992 (PMC8003469; doi:10.3390/nu13030992)
Supplement: Supplementary file 1 [file nutrients-13-00992-s001.zip › Supplementary Figure S4.pptx]

## Slide 1
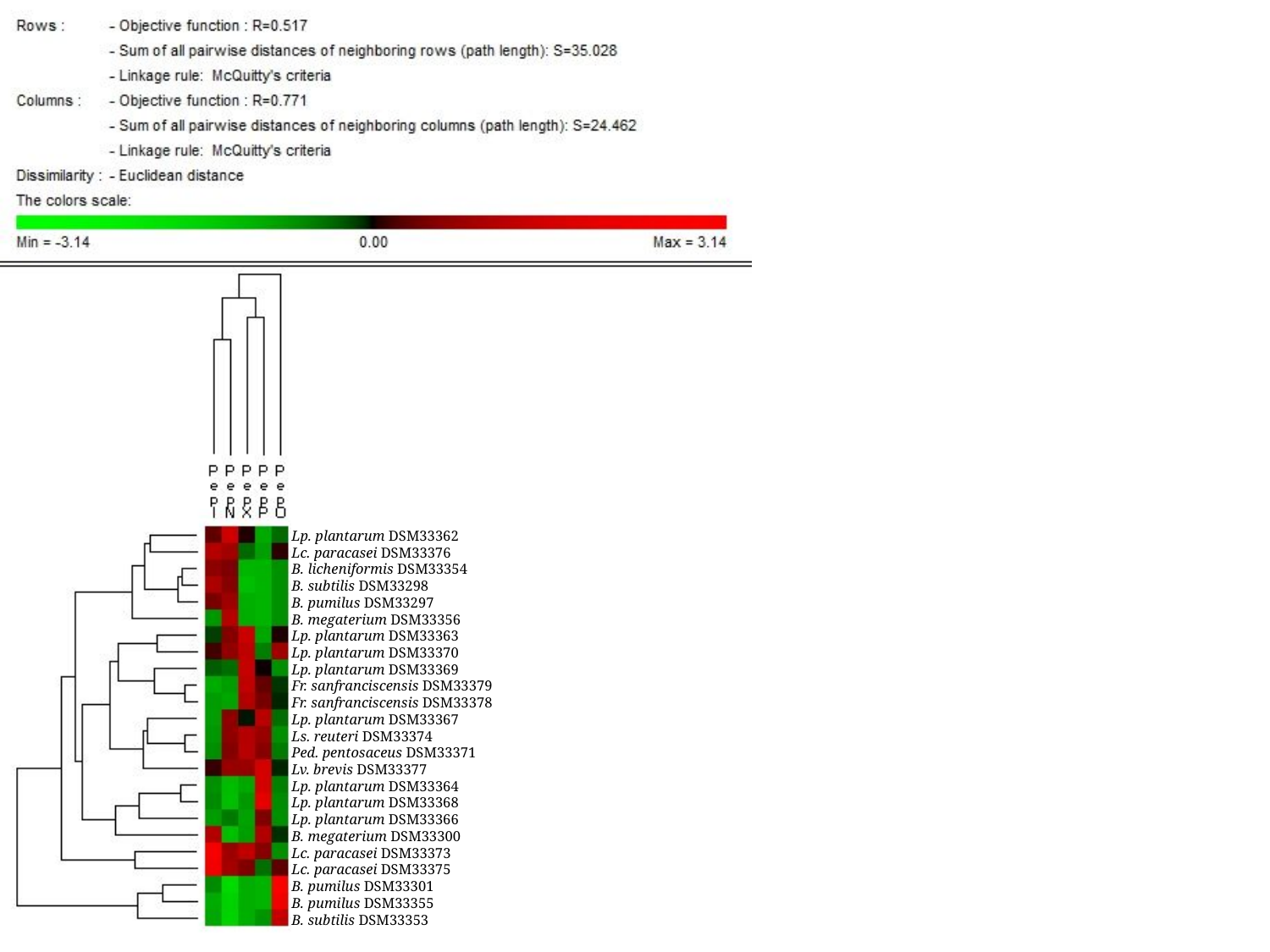

Lp. plantarum DSM33362
Lc. paracasei DSM33376
B. licheniformis DSM33354
B. subtilis DSM33298
B. pumilus DSM33297
B. megaterium DSM33356
Lp. plantarum DSM33363
Lp. plantarum DSM33370
Lp. plantarum DSM33369
Fr. sanfranciscensis DSM33379
Fr. sanfranciscensis DSM33378
Lp. plantarum DSM33367
Ls. reuteri DSM33374
Ped. pentosaceus DSM33371
Lv. brevis DSM33377
Lp. plantarum DSM33364
Lp. plantarum DSM33368
Lp. plantarum DSM33366
B. megaterium DSM33300
Lc. paracasei DSM33373
Lc. paracasei DSM33375
B. pumilus DSM33301
B. pumilus DSM33355
B. subtilis DSM33353
