# Supplementary material for: Selection of Gut-Resistant Bacteria and Construction of Microbial Consortia for Improving Gluten Digestion under Simulated Gastrointestinal Conditions
Source: Nutrients. 2021 Mar 19;13(3):992. doi: 10.3390/nu13030992 (PMC8003469; doi:10.3390/nu13030992)
Supplement: Supplementary file 1 [file nutrients-13-00992-s001.zip › Supplementary Table S1.docx]

**Supplementary Table 1.** List of the 24 out of 131 resistant bacterial strains to the gastrointestinal (GI) transit *in vitro* at different conditions [pH2, pH3, pH2 plus Skim Milk (pH2SM), and pH8], which also showed the best peptidase activities.

|  |  | **Gastric digestion (CFU log_10_)** | | | | **Intestinal digestion (CFU log_10_)** | | | |
| --- | --- | --- | --- | --- | --- | --- | --- | --- | --- |
| Species | Strain | pH2 | pH3 | pH2SM | pH8 | pH2 | pH3 | pH2SM | pH8 |
| *Bacillus licheniformis* | DSM33354 | 9±0.09 | 9.5±0.1 | 9.7±0.07 | 9.7±0.05 | 7.7±0.06 | 8.7±0.04 | 8.8±0.05 | 8.46±0.11 |
| *Bacillus megaterium* | DSM33356 | 8.3±0.08 | 8.6±0.05 | 9.5±0.1 | 9.3±0.03 | 8.3±0.06 | 8.6±0.05 | 9.5±0.12 | 9.3±0.06 |
| *Bacillus megaterium* | DSM33300 | 8±0.04 | 8.9±0.05 | 8.1±0.07 | 8.5±0.02 | 8±0.04 | 8.9±0.03 | 8.1±0.04 | 8.5±0.07 |
| *Bacillus pumilus* | DSM33297 | 6.69±0.03 | 8.18±0.11 | 8.76±0.05 | 8.76±0.07 | 6.6±0.03 | 7.11±0.05 | 7.9±0.01 | 7.85±0.06 |
| *Bacillus pumilus* | DSM33355 | 7.89±0.09 | 8.11±0.1 | 8.32±0.1 | 8.32±0.06 | 7.4±0.03 | 8.02±0.08 | 8±0.1 | 8.26±0.09 |
| *Bacillus pumilus* | DSM33301 | 8.61±0.12 | 8.7±0.09 | 9.3±0.21 | 9±0.13 | 8.4±0.06 | 8.58±0.09 | 8.7±0.12 | 8.3±0.09 |
| *Bacillus subtilis* | DSM33353 | 7.95±0.08 | 8±0.08 | 8.45±0.07 | 8.45±0.08 | 7.32±0.1 | 7.74±0.08 | 7.82±0.09 | 7.84±0.12 |
| *Bacillus subtilis* | DSM33298 | 7.78±0.07 | 7.9±0.05 | 8.13±0.13 | 8.13±0.09 | 7.32±0.06 | 7.5±0.05 | 8±0.09 | 8.52±0.11 |
| *Levilactobacillus brevis* | DSM33377 | 7.44±0.01 | 8.24±0.07 | 8.45±0.08 | 8.45±0.12 | 7.03±0.08 | 7.19±0.07 | 7.70±0.04 | 7.43±0.05 |
| *Lacticaseibacillus paracasei* | DSM33373 | 8.12±0.06 | 8.54±0.05 | 9.28±0.09 | 9.52±0.13 | 7.35±0.09 | 7.74±0.11 | 9.31±0.08 | 9.31±0.05 |
| *Lacticaseibacillus paracasei* | DSM33375 | 6.7±0.06 | 6.7±0.05 | 8.7±0.08 | 8.5±0.05 | 6.7±0.04 | 6.7±0.08 | 7.9±0.03 | 8.5±0.02 |
| *Lacticaseibacillus paracasei* | DSM33376 | 7.6±0.07 | 8.8±0.09 | 8.5±0.13 | 8.9±0.14 | 7.4±0.09 | 8.6±0.08 | 8.5±0.11 | 8.6±0.08 |
| *Lactiplantibacillus plantarum* | DSM33369 | 7.4±0.05 | 8.4±0.11 | 8.1±0.09 | 9.2±0.13 | 7±0.04 | 7±0.08 | 7.4±0.05 | 8.6±0.09 |
| *Lactiplantibacillus plantarum* | DSM33368 | 9.2±0.11 | 9.5±0.12 | 9.5±0.07 | 9.8±0.09 | 6.4±0.08 | 7.6±0.11 | 9.6±0.15 | 9.4±0.09 |
| *Lactiplantibacillus plantarum* | DSM33367 | 6.47±0.07 | 8.85±0.05 | 8.92±0.04 | 8.67±0.05 | 6±0.06 | 7.9±0.06 | 8.03±0.05 | 8.15±0.09 |
| *Lactiplantibacillus plantarum* | DSM33366 | 8.5±0.06 | 8.4±0.07 | 8.8±0.08 | 8.9±0.11 | 7.9±0.08 | 7.6±0.11 | 7.5±0.06 | 7.8±0.08 |
| *Lactiplantibacillus plantarum* | DSM33362 | 9.3±0.07 | 9.5±0.06 | 9.4±0.09 | 9.5±0.12 | 7.5±0.08 | 7.7±0.07 | 7.9±0.05 | 7.9±0.08 |
| *Lactiplantibacillus plantarum* | DSM33364 | 7.2±0.01 | 8.3±0.08 | 8.8±0.06 | 9±0.13 | 6.9±0.08 | 8.5±0.06 | 8.2±0.05 | 9.3±0.07 |
| *Lactiplantibacillus plantarum* | DSM33363 | 7.2±0.04 | 8.1±0.08 | 8.9±0.13 | 9.2±0.08 | 6.89±0.07 | 8.29±0.05 | 7.8±0.04 | 9.2±0.05 |
| *Lactiplantibacillus plantarum* | DSM33370 | 7.11±0.07 | 7.69±0.08 | 8.95±0.03 | 8.65±0.04 | 6±0.01 | 7.33±0.08 | 8.3±0.05 | 8.48±0.07 |
| *Limosilactobacillus reuteri* | DSM33374 | 7.22±0.07 | 8.56±0.05 | 9.04±0.07 | 9.13±0.09 | 7±0.06 | 8.34±0.06 | 8.4±0.04 | 8.2±0.12 |
| *Fructilactobacillus sanfranciscensis* | DSM33379 | 7.16±0.08 | 8.01±0.03 | 8.02±0.05 | 8.38±0.07 | 7.13±0.02 | 7.63±0.03 | 7.76±0.04 | 7.36±0.09 |
| *Fructilactobacillus sanfranciscensis* | DSM33378 | 8.88±0.03 | 7.99±0.07 | 8.5±0.05 | 8.5±0.08 | 7.99±0.04 | 7.87±0.08 | 8.43±0.06 | 8.48±0.05 |
| *Pediococcus pentosaceus* | DSM33371 | 7.67±0.07 | 8.99±0.03 | 8.94±0.12 | 8.36±0.11 | 6.93±0.09 | 6.99±0.08 | 8.57±0.05 | 8.58±0.03 |

Data are the mean of three independent analyses.
