# Supplementary figures and images for: Selection of Gut-Resistant Bacteria and Construction of Microbial Consortia for Improving Gluten Digestion under Simulated Gastrointestinal Conditions
Source: Nutrients. 2021 Mar 19;13(3):992. doi: 10.3390/nu13030992 (PMC8003469; doi:10.3390/nu13030992)

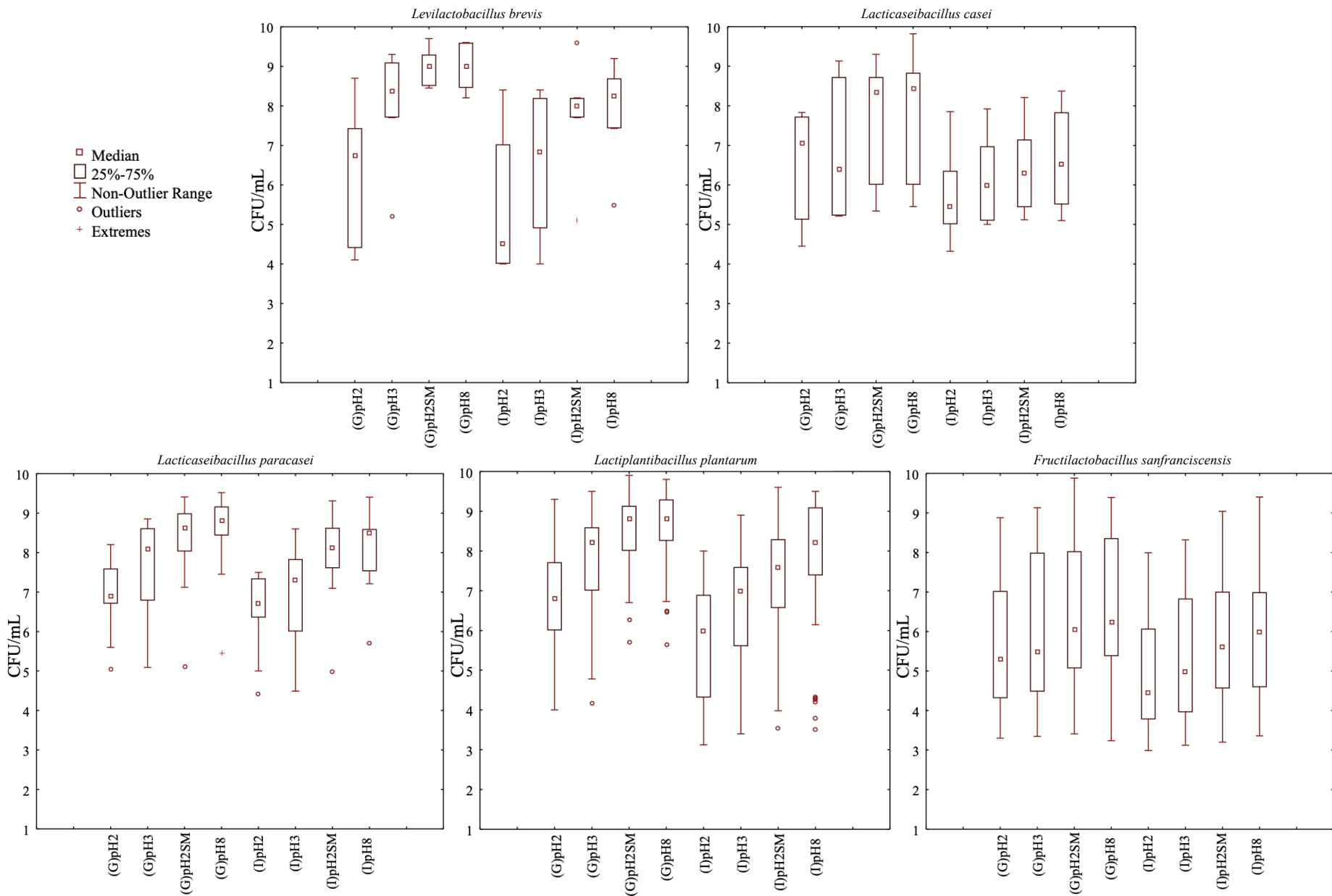

□ Median  
□ 25%-75%  
+ Non-Outlier Range  
○ Outliers  
+ Extremes

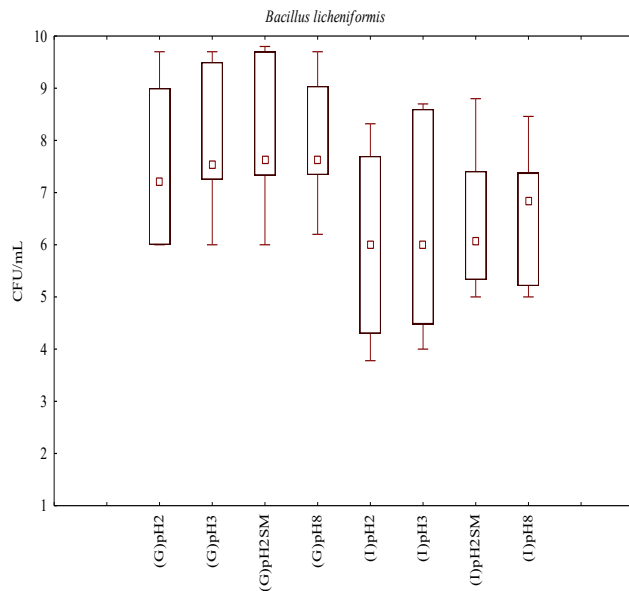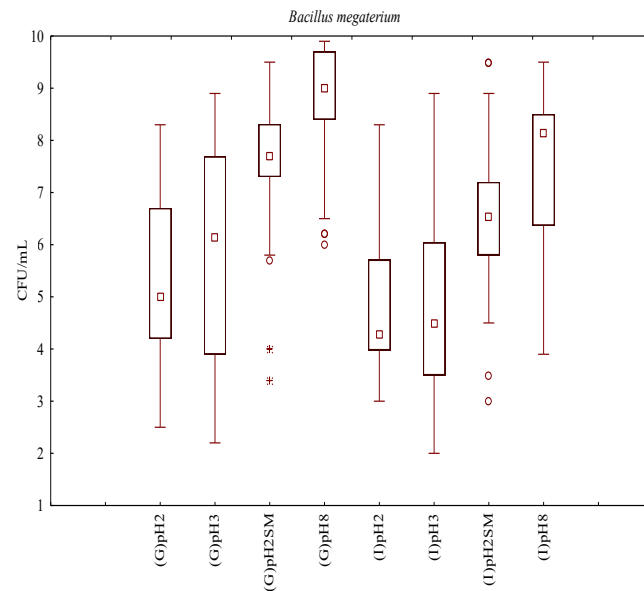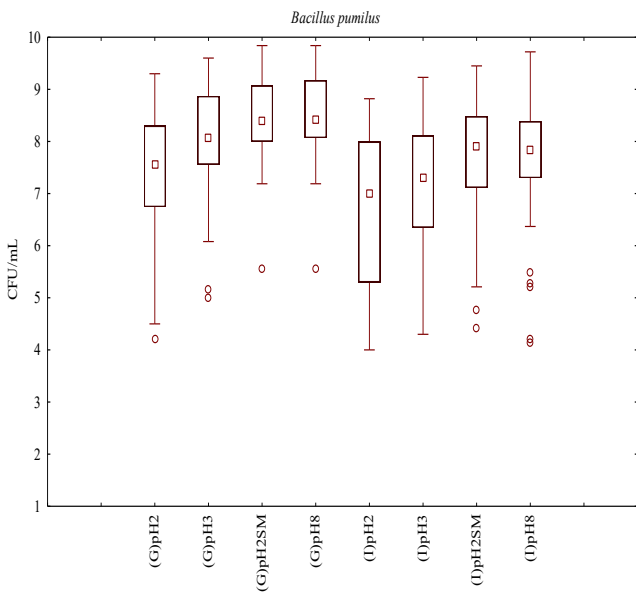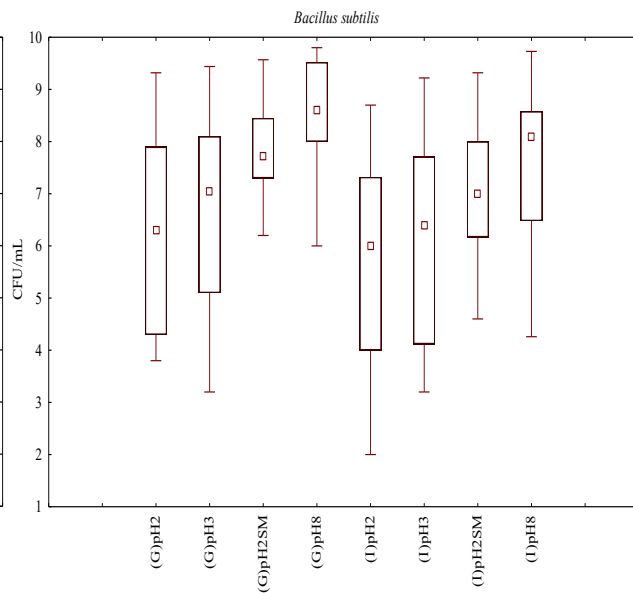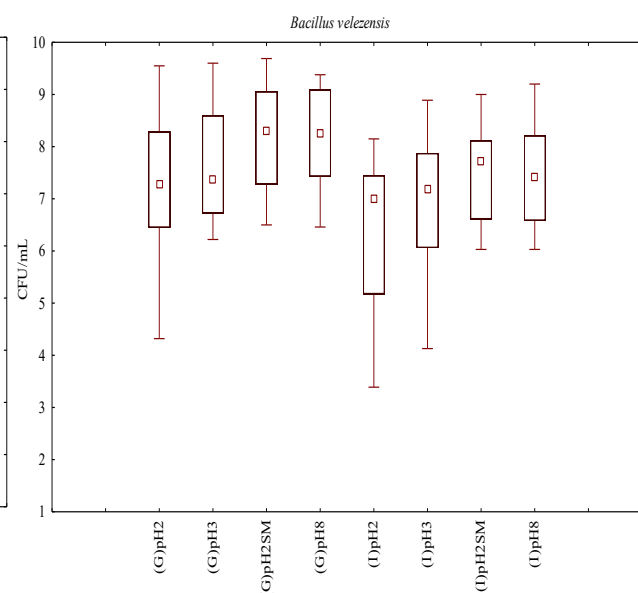

Supplement: Supplementary file 1 [file nutrients-13-00992-s001.zip › Supplementary Figure S2.pdf]

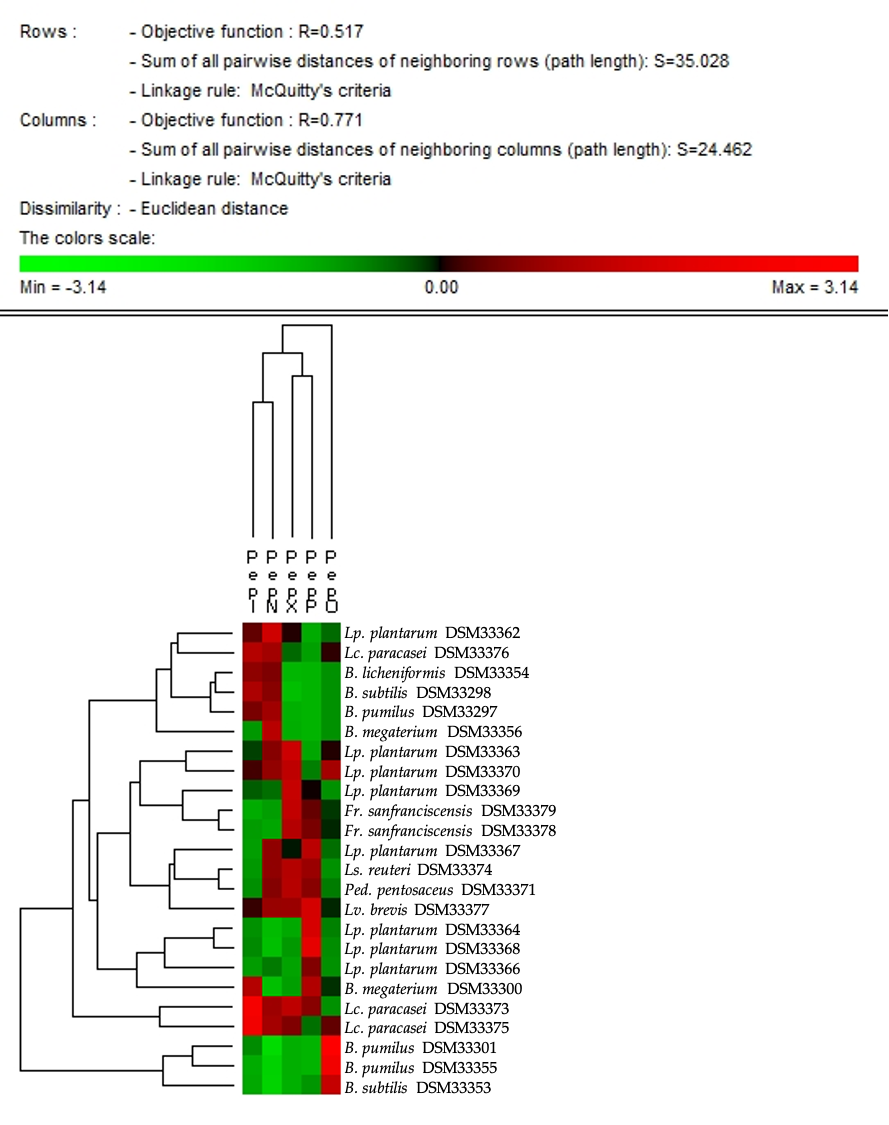

Supplement: Supplementary file 1 [file nutrients-13-00992-s001.zip › Supplementary Figure S4.png]

**A**

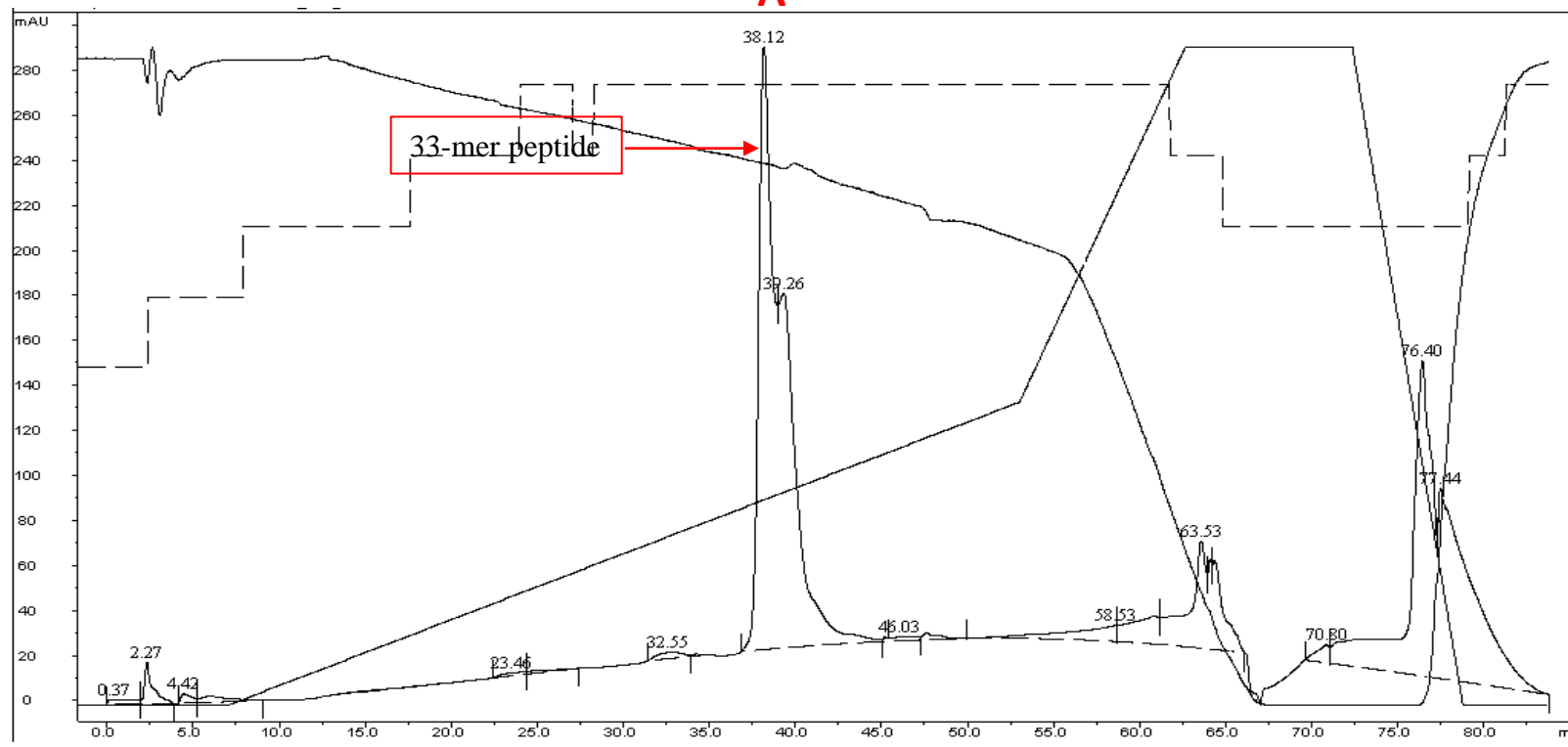

**B**

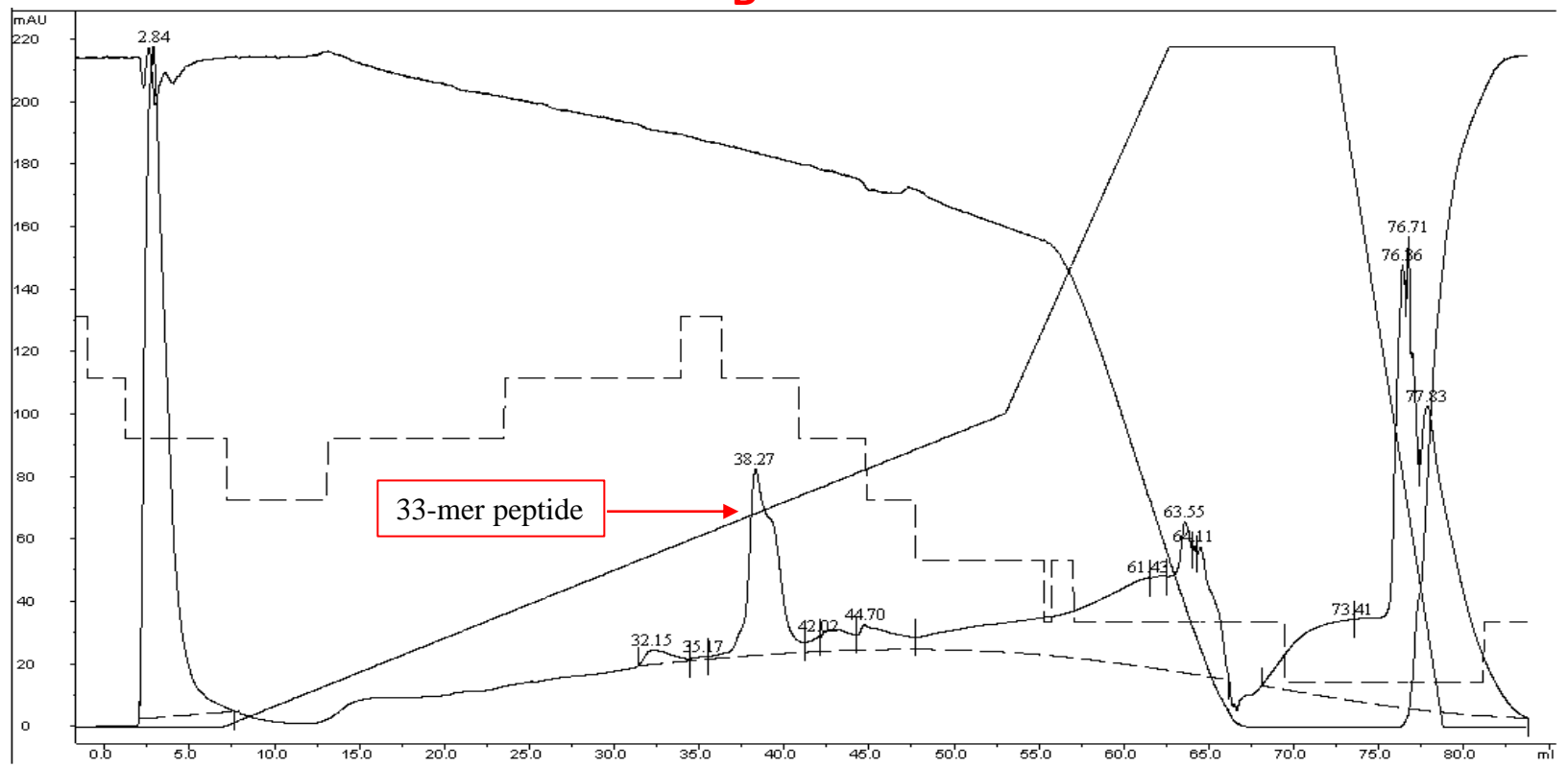

C

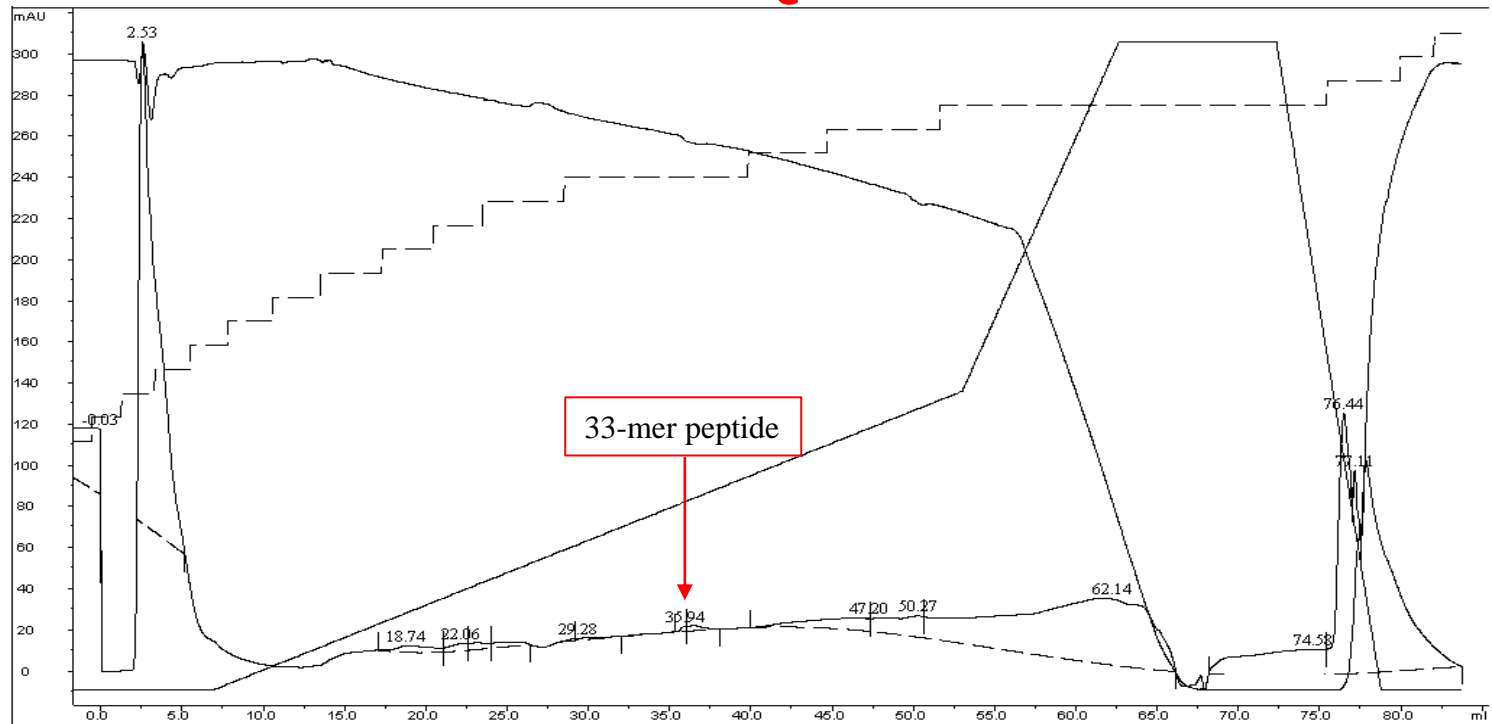

D

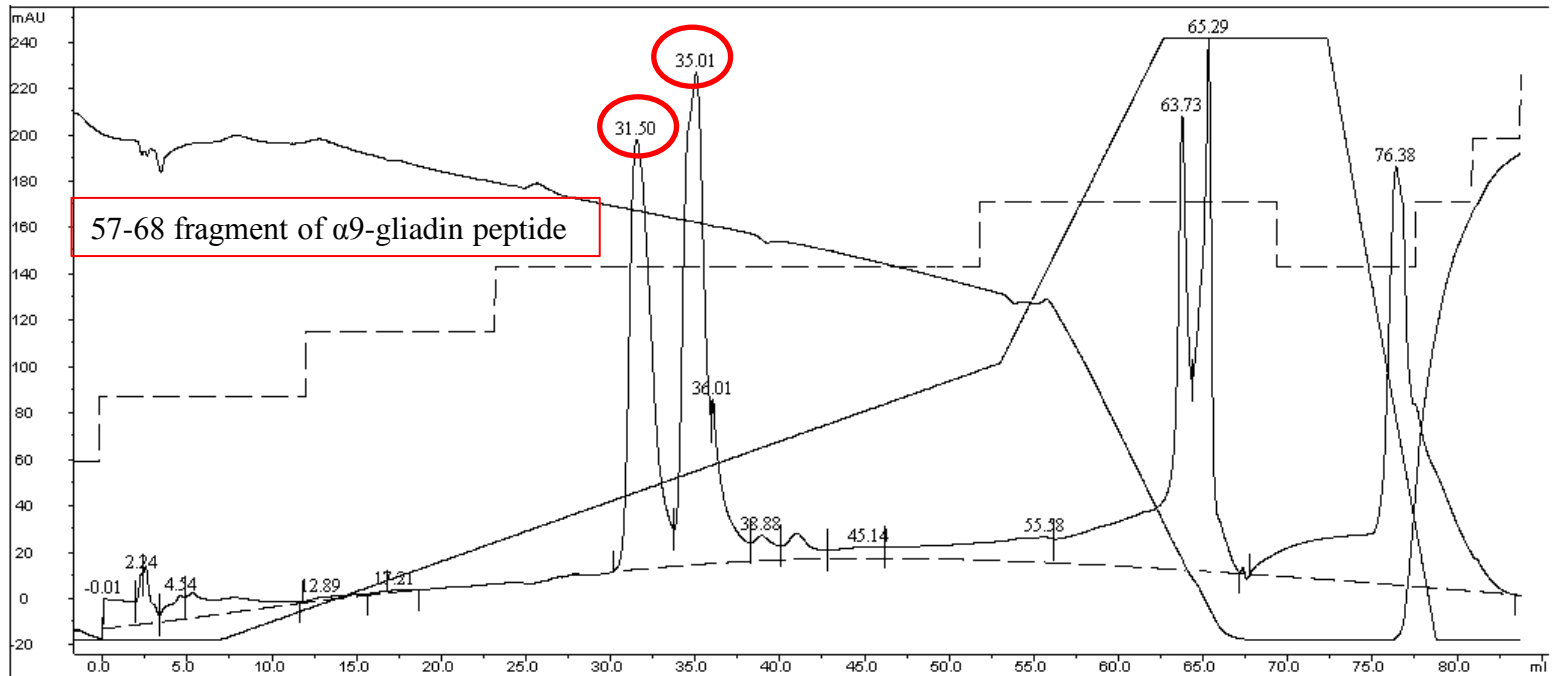

E

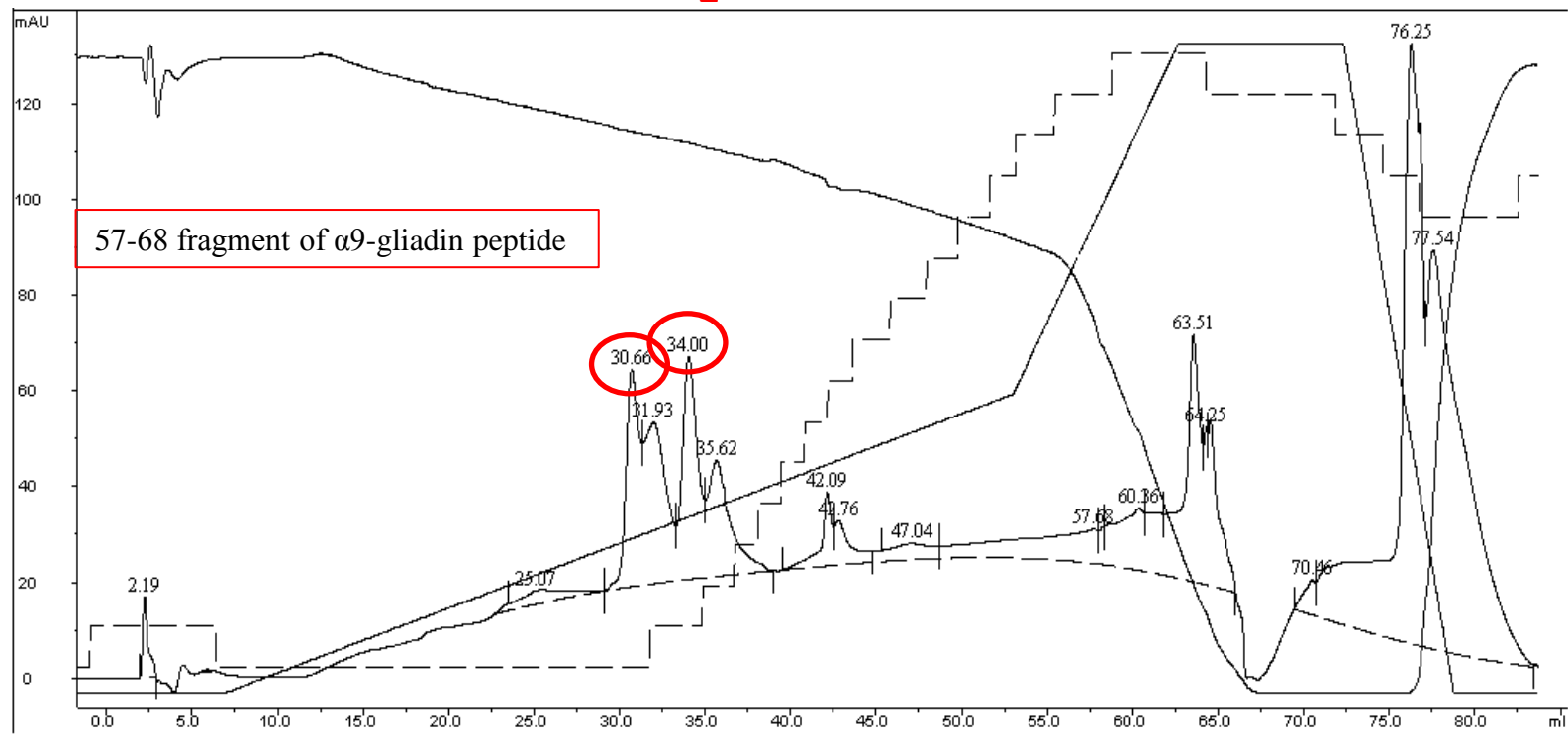

F

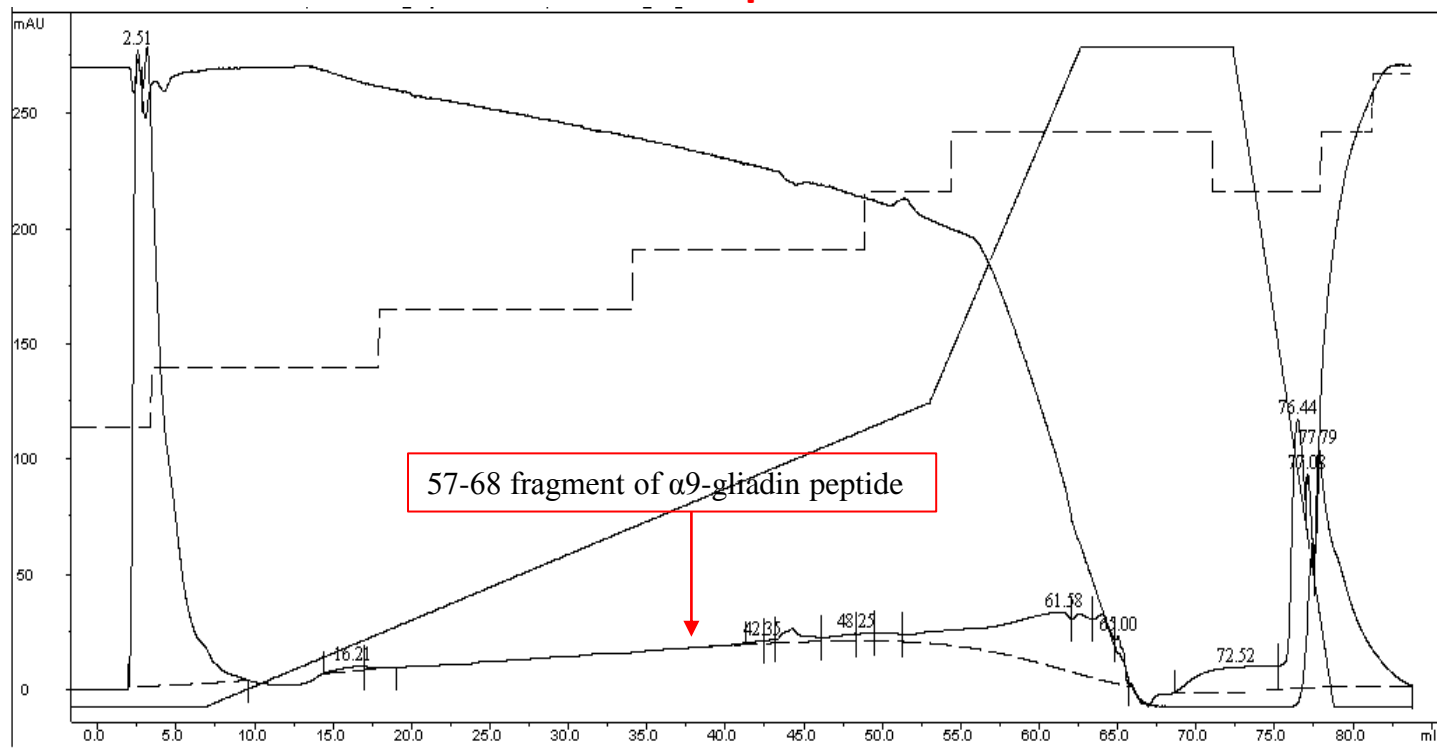

**G**

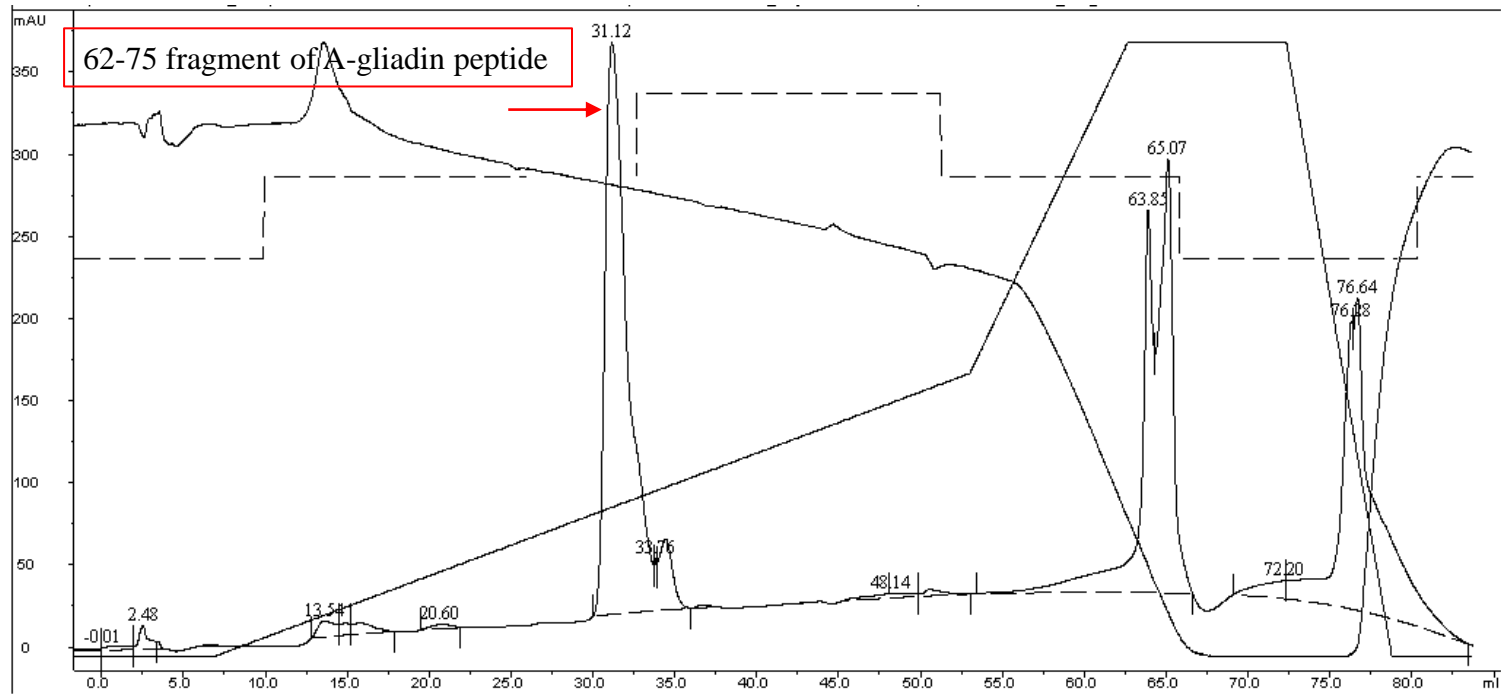

H

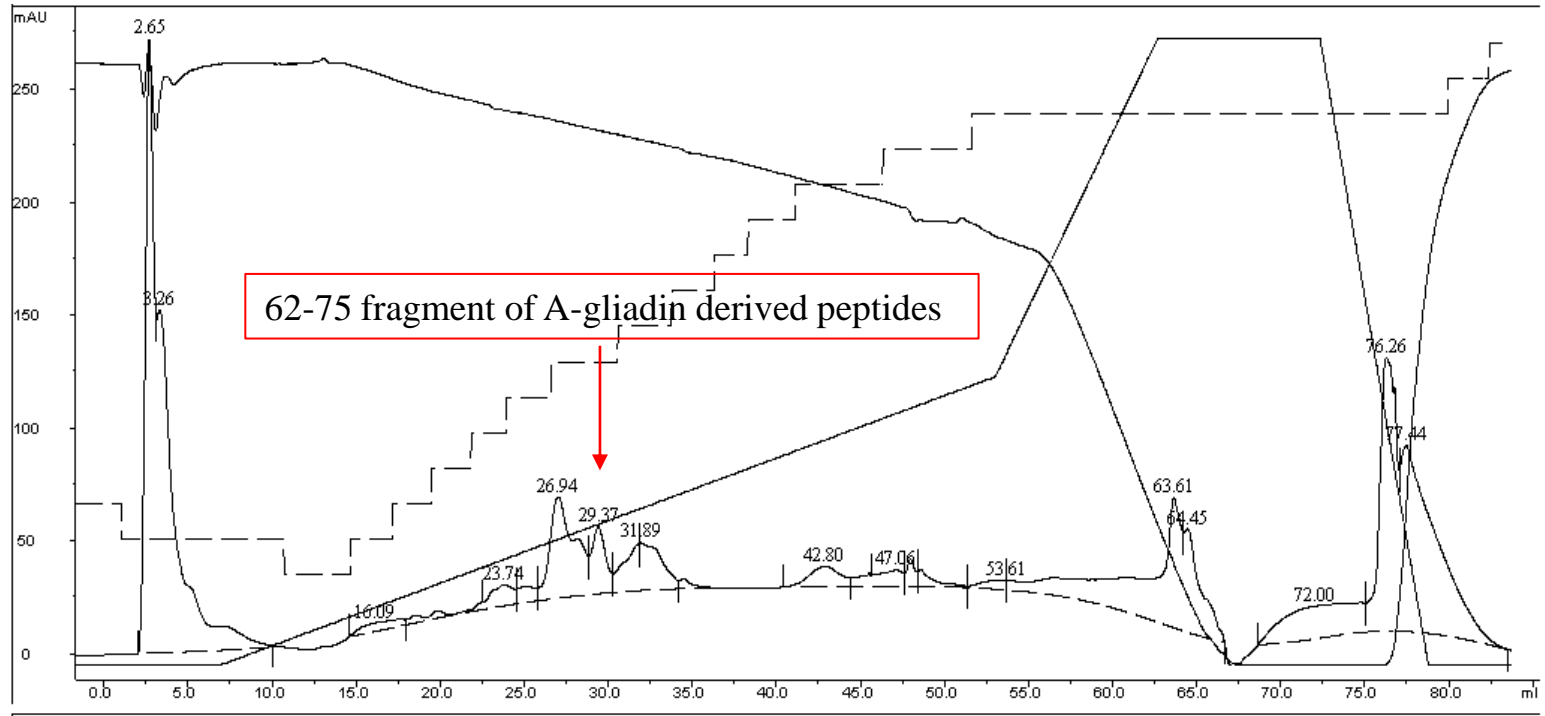

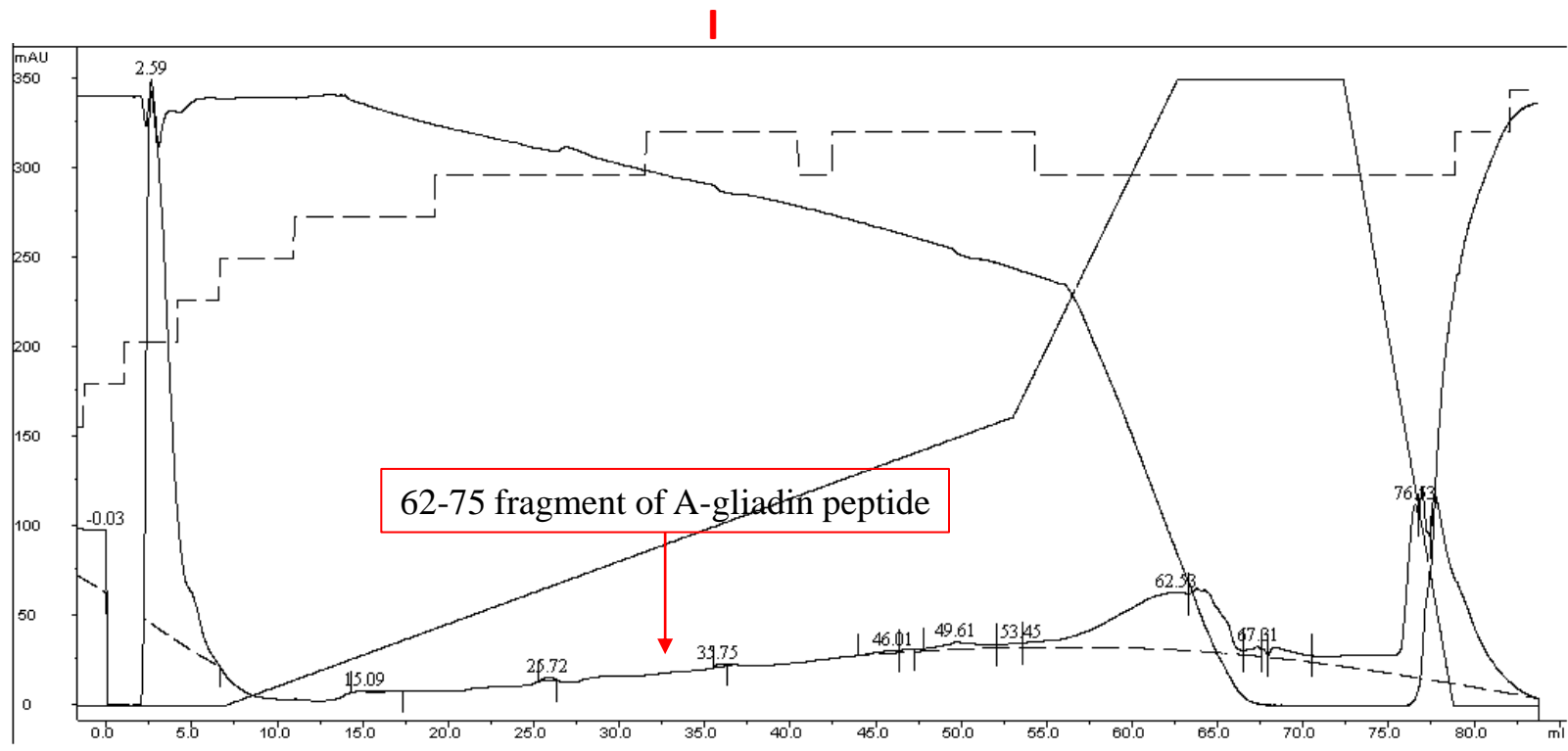

J

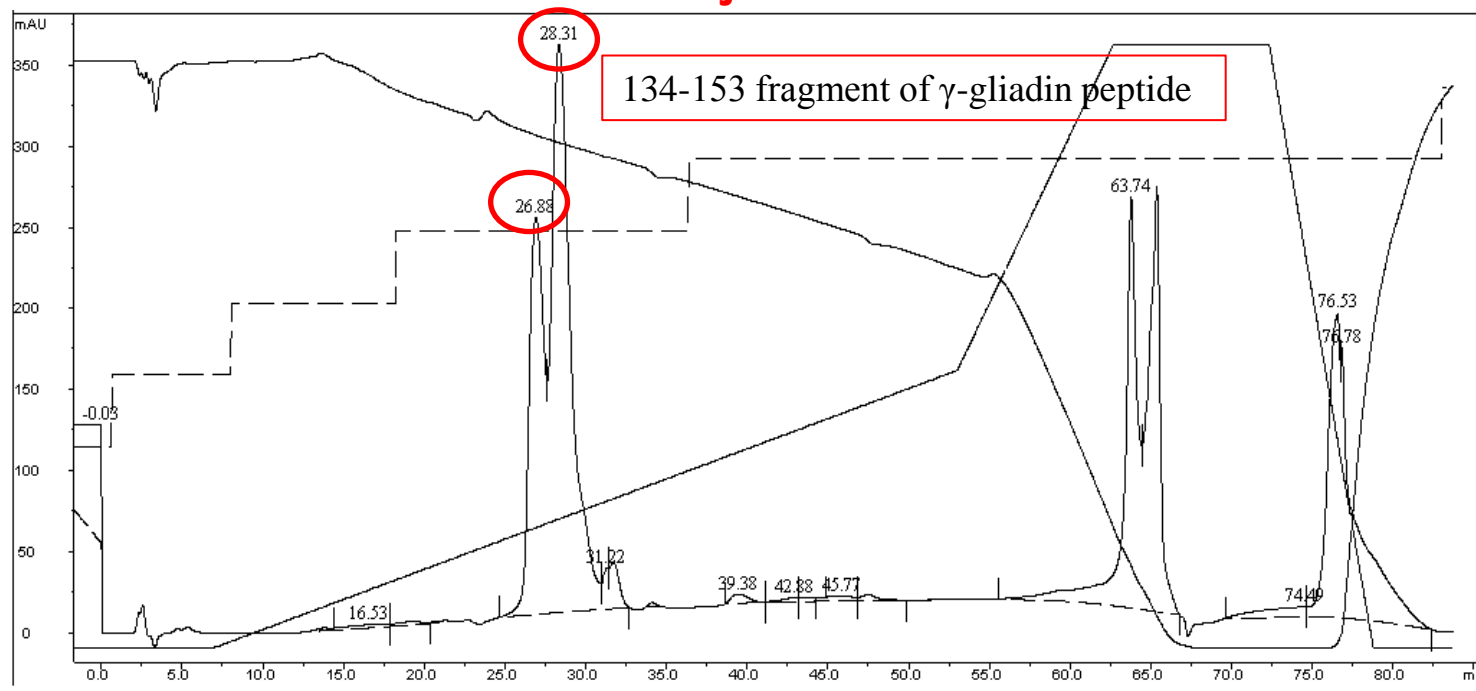

K

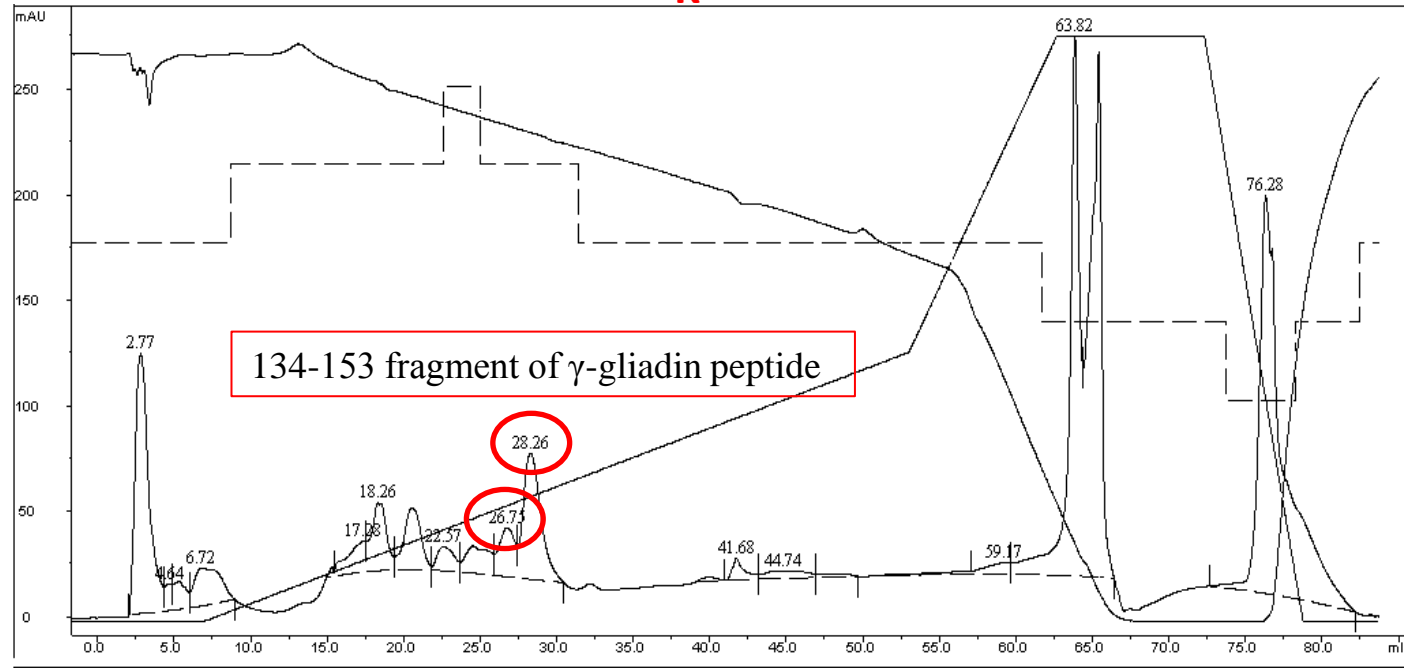

L

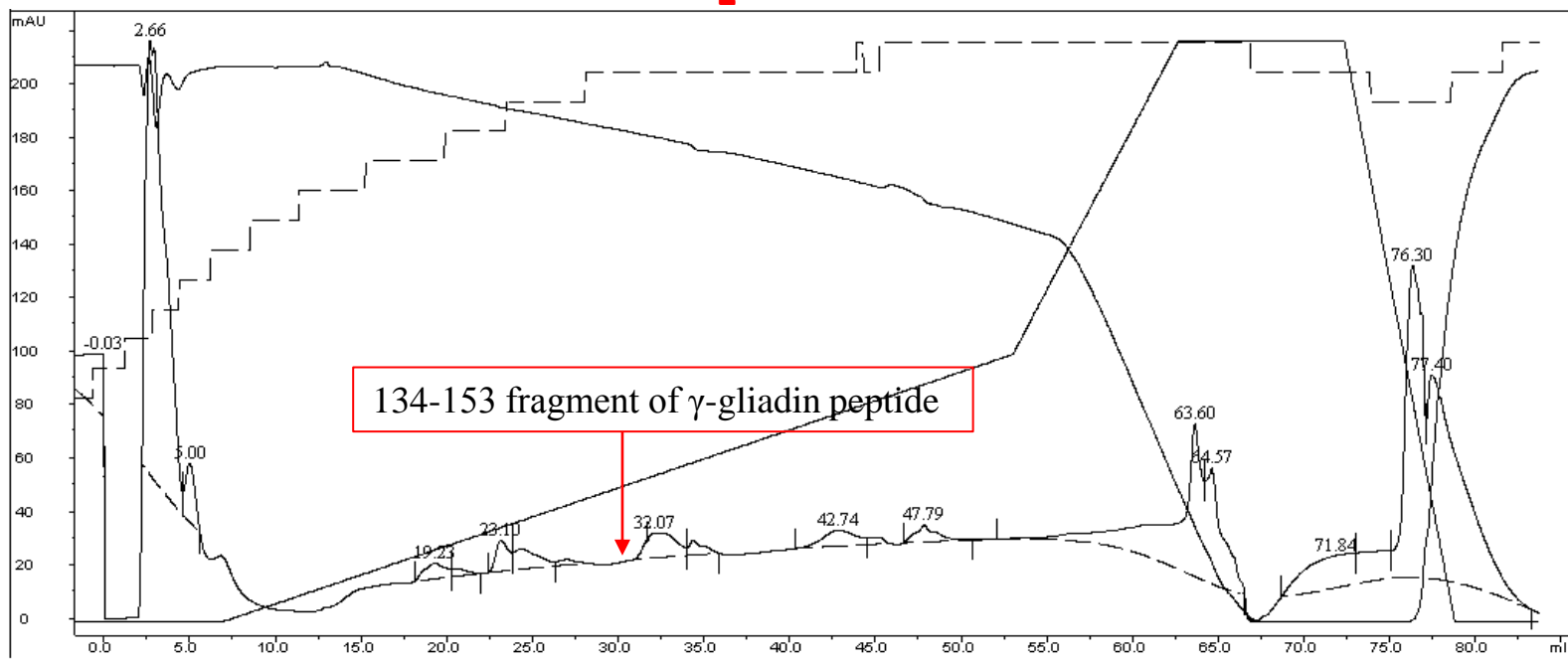

Supplement: Supplementary file 1 [file nutrients-13-00992-s001.zip › Supplementary Figure S5.pdf]

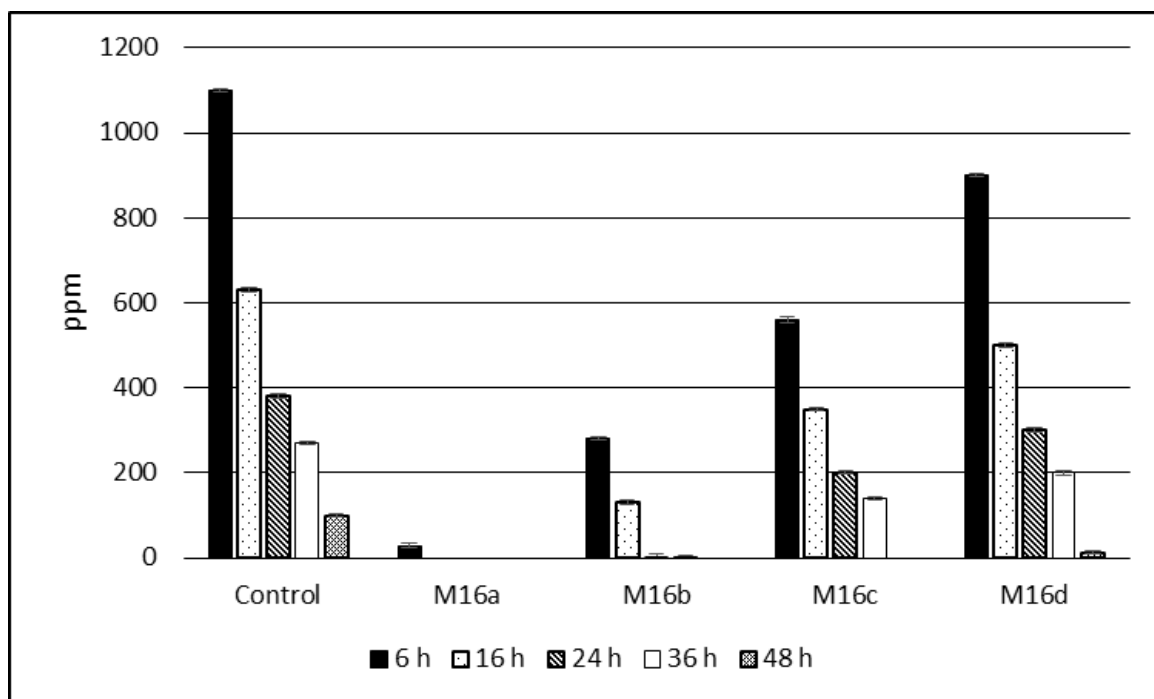

Supplement: Supplementary file 1 [file nutrients-13-00992-s001.zip › Supplementary Figure S6.pdf]
